# Sixteen Years of the Hospital Sunday Fund: Its Influence on the Metropolitan Medical Charities

**Published:** 1888-11-24

**Authors:** Sydney H. Waterlow

**Affiliations:** Treasurer of St. Bartholomew's Hospital


### Sixteen Years of the Hospital Sunday Fund;

### ITS INFLUENCE ON THE MEDICAL CHARITIES OF THE METROPOLIS.

By Sir Sydney H. Waterlow, Bart., Treasurer of St. Bartholomew's Hospital

(*Concluded from page* 101.)

A hospital proper has two great functions to perform.

*First.*—The treatment and cure of the sick poor.

*Secondly.*—The training and education in the best possible manner of the men who are to act as physicians and surgeons to the rich and poor alike in coming generations, both at home and abroad.

It is difficult to determine which of these functions is the more important. Without the medical schools, physicians and surgeons could not be properly educated. Patients would not resort to hospitals if they did not find there properly qualified medical men to treat them.

I have often thought that the public generally do not sufficiently appreciate the advantages derived by the union of the medical school with the hospital, and there are undoubtedly many poor people who are disinclined to enter hospitals, owing to the presence of medical students and to the number of doctors present in the wards. This arises from their failing to appreciate the advantages they have over the rich in this respect. The physicians and surgeons who, at great cost, are summoned to the bedside of the rich man, needing their assistance and advice, are, as a rule, the very men that perform gratuitously the largest amount of work in the relief of the sick poor in our hospitals. In both cases the patient is tended by trained and skilled nurses; but in the hospital the appliances and facilities for the treatment of the sick are necessarily far superior to those in a private house, and the surgeon or physician in charge is not only aided and assisted by competent dressers, but his work has to be performed in the presence of the junior members of the staff, who are ever on the watch, anxious to obtain the largest experience and the best professional knowledge.

As an illustration of the growing appreciation of the excellent work in our large hospitals, I should like to mention the case of an old friend of mine. He was very rich, and held a distinguished public position; his leg was accidently broken in the City; he desired the bystanders to take him at once to the nearest general hospital; he remained there nine weeks, and then went away probably more perfectly cured and with less suffering than he would have endured if he had been taken to his beautiful home at the West End. He did not forget to give a handsome benefaction to the hospital.

Referring again to the second function of all large hospitals, viz., the training and education cf medical men, I cannot resist pointing out a state of things that has always appeared to me to be a very great anomaly, viz., that Parliament should for so many years have prohibited any medical teaching in the asylums, infirmaries, or hospitals supported by the public rates, and should have thrown the responsibility of providing and maintaining the medical schools of this metropolis on the governing bodies of our large general hospitals, supported entirely by endowments and voluntary contributions.

Thirty or forty years ago it may have been not only undesirable but practically impossible to have carried on any useful and efficient medical teaching in the old workhouses, where all classes of the poor, sick, healthy, and able-bodied were cared for under the same roof; but this condition of things was generally changed by the Poor Law Act of 1867, and the Amendment Act, 1869.

Under the authority of the Act of 1867 the guardians of the poor were authorized to erect and maintain asylums or hospitals for the sick poor and the insane, and by clause 29 it was enacted that "where the asylum is provided for the reception or relief of the sick or insane, it may be used for the purposes of medical instruction and for the training of nurses."

The great benefits which would undoubtedly have been derived from this provision, if it had remained on the statute book, it is impossible for anyone at this distance of time to estimate accurately.

Unfortunately, before any one of the proposed asylums or hospitals could be completed and set to work, or any arrangement for a medical school could be tried, the Poor Law Amendment Act, 1869, was passed. Section 20 of this Act read thus:—

"20. So much of the 29th Section of the Metropolitan Poor Act, 1867, as authorises the use of any asylum for the sick or insane *for the purposes of a medical school* is hereby repealed."

The reason for this sudden change of policy I have never been able to discover. I tried to do so many years ago, when I held the position of Chairman of the Central London Sick Asylum District, and have since endeavoured to do so, but have failed.

The number of the asylums or infirmaries erected under the authority of the Act of 1867 and subsequent Acts is constantly increasing. They contain at the present time many thousand beds for the reception of persons suffering not merely from chronic diseases, but from almost every variety of complaints to which we are all, whether rich or poor, equally liable. What is far more serious, they are becoming almost the only large institutions in the metropolis for the treatment of infectious diseases, such as small-pox, scarlet fever, and typhus fever, or for the care of the insane, and are, therefore, almost the only places where the rising medical men can gain a practical knowledge and experience of the proper treatment of those diseases which are so frequently the great scourges of our population when collected in great cities.

A large number of cases are received in the wards of the Poor Law Infirmaries, supported out of the rates, which cannot be admitted to our general hospitals, owing to their chronic character and the long time they take to run their course. These cases are most important in a scientific medical point of view; they require most careful, patient diagnosis, and are frequently capable of permanent cure by discriminating treatment and the use of improved appliances. Practical experience of the various phases of this kind of disease is most important in the interests of improved medical education; but Parliament has hitherto prohibited it. I am, however, very glad to find from a report of a Select Committee of the House of Lords, issued a few days since, that there is some hope that this prohibition may soon be removed, a result which would undoubtedly not only increase the facilities and opportunities for medical instruction in the metropolis, but would raise to a still higher standard the treatment of the sick poor in the infirmaries.

It is, I think, absolutely necessary in the public interest that medical students should have every possible opportunity of studying the practical side of their profession in the wards of all large sick asylums and hospitals. If this be true, assuredly our first claim for such facilities is upon those institutions that are supported entirely by the public rates.

